# Vertebral Trabecular Bone Mechanical Properties Vary Among Functional Groups of Cetaceans

**DOI:** 10.1093/iob/obab036

**Published:** 2022-01-07

**Authors:** D N Ingle, M E Porter

**Affiliations:** Department of Biological Sciences, Florida Atlantic University, 777 Glades Road, Boca Raton, FL 33431, USA; Department of Marine Biology, Texas A&M University at Galveston, 200 Seawolf Parkway, Galveston, TX 77554, USA; Department of Biological Sciences, Florida Atlantic University, 777 Glades Road, Boca Raton, FL 33431, USA

## Abstract

Since their appearance in the fossil record 34 million years ago, modern cetaceans (dolphins, whales, and porpoises) have radiated into diverse habitats circumglobally, developing vast phenotypic variations among species. Traits such as skeletal morphology and ecologically linked behaviors denote swimming activity; trade-offs in flexibility and rigidity along the vertebral column determine patterns of caudal oscillation. Here, we categorized 10 species of cetaceans (families Delphinidae and Kogiidae; *N* = 21 animals) into functional groups based on vertebral centra morphology, swimming speeds, diving behavior, and inferred swimming patterns. We quantified trabecular bone mechanical properties (yield strength, apparent stiffness, and resilience) among functional groups and regions of the vertebral column (thoracic, lumbar, and caudal). We extracted 6 mm^3^ samples from vertebral bodies and tested them in compression in 3 orientations (rostrocaudal, dorsoventral, and mediolateral) at 2 mm min^−1^. Overall, bone from the pre-fluke/fluke boundary had the greatest yield strength and resilience, indicating that the greatest forces are translated to the tail during caudal oscillatory swimming. Group 1, composed of 5 shallow-diving delphinid species, had the greatest vertebral trabecular bone yield strength, apparent stiffness, and resilience of all functional groups. Conversely, Group 3, composed of 2 deep-diving kogiid species, had the least strong, stiff, and resilient bone, while Group 2 (3 deep-diving delphinid species) exhibited intermediate values. These data suggest that species that incorporate prolonged glides during deep descents in the water column actively swim less, and place relatively smaller loads on their vertebral columns, compared with species that execute shallower dives. We found that cetacean vertebral trabecular bone properties differed from the properties of terrestrial mammals; for every given bone strength, cetacean bone was less stiff by comparison. This relative lack of material rigidity within vertebral bone may be attributed to the non-weight-bearing locomotor modes of fully aquatic mammals.

## Spanish

Desde su aparición en el registro fósil 34 Mya, los cetáceos modernos (delfines, ballenas y marsopas) se han radiado a diversos hábitats a nivel mundial, desarrollando vastas variaciones fenotípicas entre especies. Rasgos como la morfología esquelética y los comportamientos vinculados ecológicamente denotan actividad de natación; las compensaciones en flexibilidad y rigidez a lo largo de la columna vertebral determinan los patrones de oscilación caudal. Aquí, categorizamos 10 especies de cetáceos (familias Delphinidae y Kogiidae; *N* = 21 animales) en grupos funcionales basados ​​en la morfología de los centros vertebrales, velocidades de nado, comportamiento de buceo y patrones de nado inferidos. Cuantificamos las propiedades mecánicas del hueso trabecular (límite elástico, rigidez aparente y resiliencia) entre grupos funcionales y regiones de la columna vertebral (torácica, lumbar y caudal). Extrajimos muestras de 6 mm^3^ de cuerpos vertebrales y las probamos en compresión en tres orientaciones (rostrocaudal, dorsoventral y mediolateral) a 2 mm min^-1^. En general, el hueso de la platija tuvo el mayor límite elástico y resiliencia, lo que indica que las mayores fuerzas se trasladan a la cola durante la natación oscilatoria caudal. El grupo 1, compuesto por cinco especies de delfínidos de buceo superficial, tuvo el mayor límite elástico del hueso trabecular vertebral, rigidez aparente y resiliencia de todos los grupos funcionales. Por el contrario, el Grupo 3, compuesto por dos especies de kogiidos de inmersión profunda, tenía el hueso menos fuerte, rígido y resistente, mientras que el Grupo 2 (tres especies de delfínidos de inmersión profunda) exhibió valores intermedios. Estos datos sugieren que las especies que incorporan deslizamientos prolongados durante descensos profundos en la columna de agua nadan menos activamente y colocan cargas relativamente más pequeñas en sus columnas vertebrales, en comparación con las especies que realizan inmersiones menos profundas. Encontramos que las propiedades del hueso trabecular vertebral de los cetáceos diferían de las propiedades de los mamíferos terrestres; por cada resistencia ósea dada, el hueso de cetáceo era menos rígido en comparación. Esta relativa falta de rigidez del material dentro del hueso vertebral puede atribuirse a los modos locomotores que no soportan peso de los mamíferos totalmente acuáticos.

## Introduction

Modern cetaceans (dolphins, whales, and porpoises) first appeared approximately 34 million years ago and have radiated into diverse aquatic niches. Their habitats extend from rivers to open oceans, and from the surface to depths of several thousand meters (Hoelzel 1994; Fordyce and de Muizon 2001; Laidre et al. 2004). Tracking technologies and video analyses have been previously used to investigate the movement and behavioral ecology of cetaceans, and these studies found variations among species in diving behavior and axial body displacement during swimming (Fish 1998; Skrovan et al. 1999; Williams et al. 2000; Buchholtz 2001; Rohr et al. 2002; Sveegaard et al. 2015; Calambokidis et al. 2019).

With their streamlined forms, cetaceans can descend in the water column, experiencing additions of 1 ATM of pressure for every 10 m of depth gained. Increasing pressures compress respiratory structures; during diving trials, bottlenose dolphin (*Tursiops truncatus*) alveoli collapsed at a depth of 70 m and 7 ATM (Ridgway and Howard 1979). The gradual compression of air volume in the lungs during descent correlates with animals becoming more negatively buoyant. This reduction in buoyancy results in less energy being expended by a diving cetacean due to prolonged periods of gliding and a greater ratio of gliding to active swimming (Skrovan et al. 1999; Williams et al. 2000). However, diving depths vary among cetaceans, and if bottlenose dolphin alveolar collapse depth (70 m) is representative among these aquatic mammals, then many species do not habitually reach depths where they may exercise their full gliding potential (Berta 2015).

Cetaceans are broadly categorized as caudal oscillators, a swimming mode where large forces are generated by the dorsoventral movement of a broad, lunate-shaped fluke (Fish 1993). Historically, caudal oscillators have been described as having a rigid neck (cervical vertebrae), chest (thoracic vertebrae), and anterior/central torso (posterior thoracic–anterior caudal vertebrae). The rigid fluke (posterior caudal) is attached to a narrow and flexible caudal peduncle (anterior/central caudal), and this region receives the greatest loads from the swimming muscles (Pabst 1993; Long et al. 1997; Buchholtz 2001; Buchholtz and Shur 2004). However, interspecific body displacement differences have been previously detected; for example, Buchholtz (2001) found that dorsoventral displacement began at the chest in a swimming humpback whale (*Megaptera novaeangliae*), while movement was largely restricted to the caudal peduncle in the harbor porpoise (*Phocoena phocoena*) and the Atlantic white-sided dolphin (*Lagenorhynchus acutus*).

The amount of flexibility along the vertebral column is partially due to regional variations in morphology (Table S3; Long et al. 1997; Boszczyk et al. 2001; Buchholtz 2001; Buchholtz and Shur 2004; Pierce et al. 2011; Marchesi et al. 2020a, 2020b, 2016). Rigid regions have vertebrae with disc-like centra that are taller and wider than they are long. In contrast, vertebrae with longer, spool-shaped centra indicate regions of increased flexibility, which is partially attributed to the minimal overlap of their articular processes. This reduction in overlap weakens the stiffening mechanism (Long et al. 1997; Buchholtz 2001; Buchholtz and Shur 2004; Pierce et al. 2011).

Vertebral process lengths have been described to be proportional to the mechanical force the muscles must exert to move or stabilize the vertebral column (Smith et al. 1976; Pabst 1990; Buchholtz and Shur 2004; Kardong 2009). In extant cetaceans, the longest spinous and transverse processes are in the anterior and mid-torso regions, resulting in larger surface areas for axial muscle attachment. Longer processes also restrict movement, suggesting high body stiffness in these regions (Pabst 2000; Marchesi et al. 2016).

Gross skeletal morphology, although functionally indicative of body movement, scales allometrically with growth and changes little after physical maturity has been reached (Buschang 1982; Kilborne and Makovicky 2010). By comparison, bone at the tissue level, especially trabecular bone, is highly responsive to changes in load direction and magnitude, and its structure correlates strongly with an animal's interactions with and movements in its environment (Carter and Beaupre 2001; Pontzer et al. 2006; Dumont et al. 2013; Houssaye and Botton-Divet 2018). For example, Ingle and Porter (2021) found that vertebral trabecular bone volume fraction was greater in shallow-diving delphinids compared with deep-diving delphinids, indicating differential loading and a possible variation in vertebral bone mechanical function between these groups. Additionally, Ingle and Porter (2020) found that Florida manatee (*Trichechus manatus latirostris*) vertebral trabecular bone stiffness (i.e., material rigidity) fell at the lower end of the range of terrestrial mammalian bone, which may be attributed to these aquatic mammal's lack of weight-bearing activity (Mitton et al. 1997; Borah et al. 2000). Despite ecomorphological variations in vertebral trabecular bone, the rostrocaudal orientation is considered the principal direction of stress and is a shared characteristic among mammals spanning both aquatic and terrestrial environments (Mosekilde and Mosekilde 1986; Smit et al. 1997; Smit 2002; Aiyanger et al. 2014; Ingle and Porter 2020).

Creating functional groups, or ecomorphological designations, can be useful to examine drivers for functional and morphological variation among closely related species (Buchholtz 2001; Dumont et al. 2013). Here, we designate 10 species of dolphins and small whales (order: Cetacea; parvorder: Odontocete; families: Delphinidae and Kogiidae) into three functional groups. These groups were based on vertebral centra morphology and diving behavior reported in previous literature, in which vertebral trabecular bone microarchitecture was assessed from the same individuals used in this study (Ingle and Porter 2021).

### Groups 1 and 2

These odontocetes (family Delphinidae) were defined by lumbar vertebral centum lengths that were either equal or less than their widths and heights in lumbar vertebrae, with a drastic shortening of centra at the central/posterior caudal boundary, while relatively longer centra were found in the posterior thoracic region and anterior caudal peduncle (Buchholtz 2001). This vertebral morphology is indicative of fast swimming and restricted bending in the rigid torso. Buchholtz (2001) found that axial body bending in a swimming Atlantic white-sided dolphin (*Lagenorhynchus acutus*) was largely restricted to the caudal peduncle and fluke. Group 1 and Group 2 species were separated by habitual diving behavior. Group 1 species have average dive depths that do not exceed 50 m, which is shallower than the depth at which the collapse of the respiratory system occurs (Ridgway and Howard 1979; Skrovan et al. 1999). In comparison, Group 2 delphinids can habitually dive to 100 m, beyond the point of alveolar collapse (Buchholtz 2001). Once the respiratory system collapses, animals become negatively buoyant and can descend through the water column with a sinking glide (Ridgway and Howard 1979; Skrovan et al. 1999). Deep divers may spend a greater percentage of their time gliding than their counterparts that forage in shallow waters.

### Group 3

These cetaceans (families Kogiidae, Physeteridae, and Balaenopteridae) had nearly consistent centrum lengths, widths, and heights throughout the torso, with a relative shortening of centra at the central/posterior caudal region (Buchholtz 2001). This vertebral morphology suggests a flexible body axis; in a swimming humpback whale (*Megaptera novaeangliae*; family Balaenopteridae), undulation occurred throughout the torso and was interrupted at the fluke, a stiff structure that produces thrust (Buchholtz 2001; Gough et al. 2018). Similar to Group 2, species in Group 3 regularly forage at depths greater than 100 m, where habitual respiratory system collapse may occur.

Here, we aim to determine the vertebral trabecular bone mechanics of ten cetacean species (families: Delphinidae and Kogiidae) with different vertebral morphologies and diving behaviors. We quantified the mechanical properties of these vertebrae to understand stress at permanent deformation (yield strength, σ_y_), material rigidity (apparent stiffness, *E*_app_), and the ability to absorb energy (resilience, *U*_r_). We examined these properties among (1) three functional groups (Groups 1–3) as determined by vertebral morphology and diving depth, (2) vertebral column regions (posterior thoracic, lumbar, anterior caudal, and central/posterior caudal), and (3) testing orientations (rostrocaudal, dorsoventral, and mediolateral). Additionally, we examined (4) the relationships between bone mechanical properties and vertebral process lengths and (5) compared mechanical properties with previous literature on aquatic and terrestrial mammals.

We hypothesized that all three vertebral trabecular bone mechanical properties (yield strength, apparent stiffness, and resilience) would be greatest in delphinid species with average diving depths of less than 70 m (Group 1), who may actively swim a greater proportion of the time compared with their deep-diving counterparts (Group 2) and deep-diving kogiids (Group 3). Based on vertebral morphology, we also predicted that mechanical properties of the vertebrae from delphinid species would vary along the vertebral column. As the principal direction of stress, we expected the greatest bone yield strength, apparent stiffness, and resilience in the rostrocaudal orientation. We hypothesized that among vertebral bone supporting axes (non-principal directions of stress; dorsoventral and mediolateral), mechanical properties of dorsoventrally and mediolaterally tested bone would positively correlate with vertebral process lengths (spinous processes and transverse processes, respectively), because longer processes may provide a greater surface area for muscle attachment. Finally, we predicted that cetacean bone mechanical properties would differ from those of terrestrial mammals, but would be similar to Florida manatees, since they share an aquatic habitat and a non-weight-bearing locomotor mode.

## Materials and methods

Vertebrae used in this study were obtained from necropsies performed at: Mote Marine Laboratory and Aquarium (Sarasota, FL, USA), Harbor Branch Oceanographic Institute Necropsy Laboratory (Fort Pierce, FL, USA), and National Oceanic and Atmospheric Administration—National Marine Fisheries Service (NMFS; Key Biscayne, FL, and Baton Rouge, LA, USA). We obtained a letter of authorization for this work from the NMFS, which was also approved by an Institutional Animal Care and Use tissue protocol from Florida Atlantic University (A(T)17-08).

### Functional groups

#### Group 1 species

The five species (family Delphinidae; Table S1) in this ecomorphological designation include: *Grampus griseus* (Risso's dolphin; *N* = 1 animal; Wells et al. 2009), *Stenella attenuata* (Pantropical spotted dolphin; *N* = 1 animal; Scott and Chivers 2009), *Stenella frontalis* (Atlantic spotted dolphin; *N* = 2 animals; Davis et al. 1996), *Steno bredanensis* (rough-toothed dolphin; *N* = 1 animal; Watkins et al. 1987), and *Tursiops truncatus* (bottlenose dolphin; *N* = 3 animals; Skrovan et al. 1999; Klatsky et al. 2007).

#### Group 2 species

Group 2 delphinids include *Feresa attenuata* (pygmy killer whale; *N* = 1 animal; Table S1; Pulis et al. 2018), *Peponocephala electra* (melon-headed whale; *N* = 4 animals; Joyce et al. 2017), and *Stenella longirostris* (spinner dolphin; *N* = 2 animals; Wursig et al. 1994; Dolar et al. 2003).

#### Group 3 species

The two species in this group include (family Kogiidae): *Kogia breviceps* (pygmy sperm whale; *N* = 5 animals) and *Kogia sima* (dwarf sperm whale; *N* = 1 animal; Table S1; Mullin et al. 1994; Mullin and Hansen 1999; McAlpine 2018).

### Vertebral dissection and preparation

We dissected vertebrae from subadult and adult animals, or individuals that were at least three years old based on species-specific body growth curves (Perrin et al. 1976; Perrin et al. 1977; Bossart et al. 1985; Caldwell and Caldwell 1989; Stolen et al. 2002; Amano and Miyazaki 2004; Siciliano et al. 2007; Amano et al. 2014; McAlpine 2018). A potential limitation to our study was that we did not examine if any ontogenetic variations were present, but previous research has shown that bone properties from another fully aquatic mammal, Florida manatees (*T. manatus latirostris*), did not vary between subadult and adult developmental stages (Ingle and Porter 2020). However, this assumption is limited, because vertebral bone structure does vary between manatees (i.e., sirenians) and cetaceans (Dumont et al. 2013).

We sampled vertebrae from the thoracic (*N* = 3), lumbar (*N* = 3), and caudal regions (*N* = 6) from a total of 21 animals, for which vertebral formulas vary interspecifically and even intraspecifically (Table 1; Pinedo 1987; Rommel 1990; Tinker 1988; Perrin and Hohn 1994; Chantrapornsyl 1996; Mignucci-Giannoni et al. 1998; Perrin 1998; Perrin 2002; Villegas-Zurita 2015). The combination of the posterior thoracic, lumbar, anterior caudal, and central caudal regions is referred to as the torso (Buchholtz 2001). For all individuals, the last three vertebrae of the thoracic series were selected for dissection. If thoracic vertebrae had been previously separated and their count was unknown, we selected bones with characters indicative of a posterior placement within the series, including a decrease in zygapophysis size and centrum dimensions similar to anterior lumbar vertebrae (Table S3; Buchholtz 2001). Lumbar vertebrae 7–9 were consistently sampled for all individuals; because kogiids have fewer lumbar vertebrae than delphinids, this selection represented a relatively more posterior location in the torso of kogiids (Table 1; Buchholtz 2001; Gillet et al. 2019).

**Table 1:** Vertebral formulas of delphinid and kogiid species sampled in the present study

Species	Common name	Thoracic	Lumbar	Caudal	References
Delphinidae (family)					
*Feresa attenuata* (1)	pygmy killer whale	13	16	33	Chantrapornsyl 1996
*Grampus griseus* (1)	Risso's dolphin	12–13	18–19	30–31	Tinker 1988
*Peponocephala electra* (4)	melon-headed whale	12	17	44	Mignucci-Giannoni et al. 1998
*Stenella attenuata* (1)	Pantropical spotted dolphin	16	20	37	Perrin and Hohn 1994
*Stenella frontalis* (2)	Atlantic spotted dolphin	13–15	15–20	28–35	Perrin 2002
*Stenella longirostris* (2)	spinner dolphin	15	18	22	Perrin 1998
*Steno bredanensis* (1)	rough-toothed dolphin	13–14	16	29	Villegas-Zurita 2015
*Tursiops truncatus* (3)	bottlenose dolphin	12–14	17–19	23–28	Rommel 1990
Kogiidae (family)					
*Kogia breviceps* (5)	pygmy sperm whale	13	9	27	Tinker 1988
*Kogia sima* (1)	dwarf sperm whale	13	10	25	Pinedo 1987

All species have seven cervical vertebrae. Numbers in parentheses after species names denote the number of individuals of that species sampled.

**Fig. 1:**
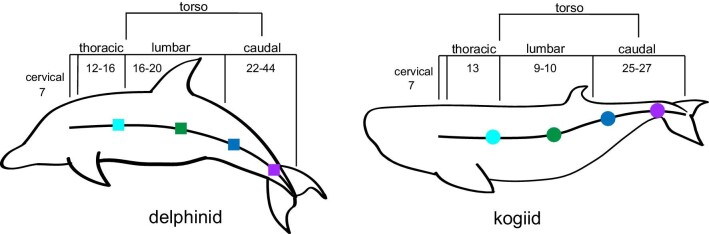
Vertebral series and regional sampling along the vertebral column of delphinids and kogiids. In both cetacean families, the vertebral column consists of a cervical, thoracic, lumbar, and caudal series. The range of vertebral counts for each series is listed for the delphinid and kogiid species sampled for the present study. The torso extends between the location of posterior thoracic vertebrae and central caudal vertebrae. Vertebrae were sampled from the posterior thoracic (light blue), lumbar (green), anterior caudal (dark blue), and central/posterior caudal (purple) locations.

For each species, caudal vertebrae (anterior caudal and central/posterior caudal regions) were selected based on body landmarks. Samples from the anterior caudal region were based on the middle centrum's alignment with the anus and the vertebrae that were immediately cranial and caudal. The central/posterior caudal boundary was identified as the first vertebra of the fluke (i.e., fluke insertion/ball vertebra) and the two cranially adjacent vertebrae (Fig. 1; Buchholtz and Shur 2004; Fish and Lauder 2006). We selected vertebrae cranial to the “ball vertebrae” because the two vertebrae caudal to this location were too small to extract samples for mechanical testing. For all individuals within a species, the same vertebrae were sampled from subsequent animals when possible. Vertebral segments were stored on ice during transport to the lab (Florida Atlantic University, Boca Raton, FL, USA). Protocols for vertebra storage and cleaning are detailed in Ingle and Porter (2020).

**Fig. 2:**
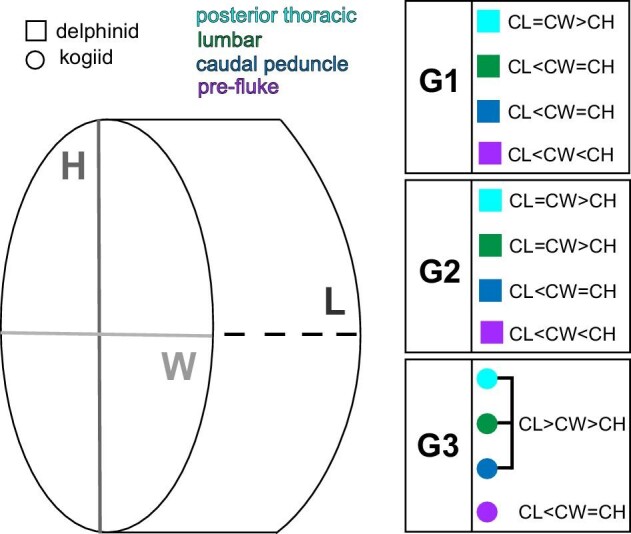
Vertebral centra dimensions among functional groups. Centra dimensions changed along the vertebral column in both Group 1 (G1; shallow-diving delphinids) and Group 2 (G2; deep-diving delphinids) species, although centra from the lumbar region in G1 species had smaller lengths than widths and heights, while G2 lumbar centra had smaller heights than lengths and widths. Group 3 (G3; deep-diving kogiids) centra dimensions were retained from the posterior thoracic to the anterior caudal region. CL = centrum length, CW = centrum width, and CH = centrum height.

For each vertebra, centrum dimensions [length (CL), width (CW), and height (CH)] and process lengths (spinous and both right and left transverses) were measured (mm; Fig. 2; Tables S2 and S3). We calculated relative centrum length, which takes into account the entire centrum's dimension, by dividing centrum length by the sum of centrum width and centrum height, or 2CL/(CW + CH) (Fig. 2; Table S3; Buchholtz 2001). Right and left transverse process lengths were averaged, unless one side was broken or damaged; no damaged process measurements were included (Table S2). Most vertebrae in the central/posterior caudal boundary lacked processes and these length measurements are absent (Table S2; Buchholtz and Shur 2004).

We found that relationships among delphinid (Groups 1 and 2) and kogiid (Group 3) CL, CW, and CH were similar to Buchholtz's (2001) observations on delphinids and physeterids, respectively. Group 1 relative centrum lengths along the vertebral column were also similar, although Group 3 relative centrum lengths were higher overall (Fig. 2; Table S3; Buchholtz 2001). It is important to note that while Buchholtz (2001) measured all vertebrae from study specimens, our data only represents “snapshots” along the column; we only measured three vertebrae from each functional region.

To extract samples for mechanical testing, we used a band saw to remove processes from vertebral bodies. We then cut a 6-mm slice in the frontal plane from the center of each centrum, and sawed several cubes (*n* = 1–5, dependent on the size of the vertebra) with 6 mm^3^ dimensions from the slice (Fig. S1). Centra dissected from the central/posterior caudal region were often too small to produce five cubes. Thus, we extracted as many cubes as possible from these vertebral slices (Ingle and Porter 2020). In total, we cut 1032 cubes from the posterior thoracic (*N* = 249), lumbar (*N* = 295), anterior caudal (*N* = 276), and central/posterior caudal (*N* = 212) regions from 21 animals representing 10 species (Ingle and Porter 2020).

### Mechanical testing

We measured the dimensions of length, width, and height (mm) from individual trabecular bone cubes with digital calipers and stored them as described in Ingle and Porter (2020). Based on *in situ* orientation, we tested cubes (1–5, depending on specimen size) from the first vertebra of each regional series (three adjacent vertebrae) rostrocaudally, the second dorsoventrally, and the third mediolaterally.

Quasi-static compression tests were conducted with a 2 kN load cell in a closed room at a temperature of 21°C (Birnbaum et al. 2002). Bone cube samples were kept moist in mammalian Ringer's solution until mechanical testing, during which they were not immersed in solution. Samples were placed directly on a stationary platen (without oil or lubricant) and the upper platen, attached to an actuator, was slowly lowered until a preload of 5 N was reached. This force is within the preload range previously described in the literature for trabecular bone (Røhl et al. 1991; Vivanco et al. 2013). After the preload cycle, samples were tested in compression through yield point at a displacement rate of 2 mm min^−1^ (Fig. S2; An and Draughn 1999; Inceoglu et al. 2006).

Mechanical properties were calculated using Bluehill Universal Software v.3.67 (Instron, Norwood, MA, USA) (An and Draughn 1999). We converted force–displacement curves to engineering stress–strain (σ–ε) curves to determine mechanical properties. We calculated apparent stiffness (*E*_app_: apparent Young's Modulus), or the resistance to compression, as the slope of the steepest point of the linear portion of the σ–ε curve (Bayraktar et al. 2004; Aiyanger et al. 2014). Here, the linear portion of the curve was divided into six regions, and the region with the steepest slope was identified and used for the apparent stiffness calculation. Yield strength (σ_y_) was determined as the point where σ of the bone cube transitions from elastic to plastic deformation (when permanent deformation occurs) and was calculated using the 0.2% offset method (Keaveny et al. 2001). Based on previous trabecular bone mechanical property studies, we focused on σ_y_ and did not measure ultimate strength (σ_ult_) (Bevill, Easley and Keaveny 2007; Wang et al. 2015). We think that measuring σ_y_, the point of bone permanent deformation, is more biologically relevant for quantifying cetacean bone behavior because they are likely not experiencing tissue failure (σ_ult_) during routine swimming. Modulus of resilience (*U*_r_) was quantified as the area under the curve to the yield point; this property measures the maximum energy that can be absorbed per unit volume without creating a permanent distortion (Sierpowska et al. 2005).

### Statistical analyses

We used three-way analysis of variance (ANOVA) models to examine differences in bone yield strength (σ_y_), apparent stiffness (*E*_app_), and resilience (*U*_r_) using the three functional groups, region (posterior thoracic, lumbar, anterior caudal, and central/posterior caudal; Fig. 1), and testing orientation (rostrocaudal, dorsoventral, and mediolateral; Fig. S1) as main effects. For each mechanical property, each vertebra is represented as the mean from mechanical tests on individual bone cubes (*n* = 1–5). For species represented by more than one animal, we averaged bone mechanical property values for each of the 12 vertebrae sampled along the column. Reporting the mean for every vertebra for all species reduces potential skewing due to unequal sample availability in our ANOVA results. Based on our hypotheses, we examined the functional group*region interaction term in our statistical models. *Post hoc* Tukey tests were conducted to examine differences among significant effects.

Next, we examined six general linear models (GLMs) to interpret the relationships among yield strength, apparent stiffness, and resilience with vertebral processes length. For each mechanical property, we used a GLM to examine data from bone tested in the dorsoventral orientation paired with spinous process length, and bone tested in the mediolateral orientation paired with transverse process length (average of right and left sides). Each GLM included animal length and vertebral process length as effects.

Statistical tests were performed using JMP v.5.0.1.a (SAS Institute Inc., Cary, NC, USA) and significance was assigned as *P* < 0.05. While statistical tests used the mean mechanical properties from each vertebra, our figures show data from each mechanical test (*n* = 1–5 cubes per vertebra). Therefore, we show the true range (error bars) of each property for cetacean trabecular bone.

## Results

### Yield strength (σ_y_) of vertebral bone

**Fig. 3:**
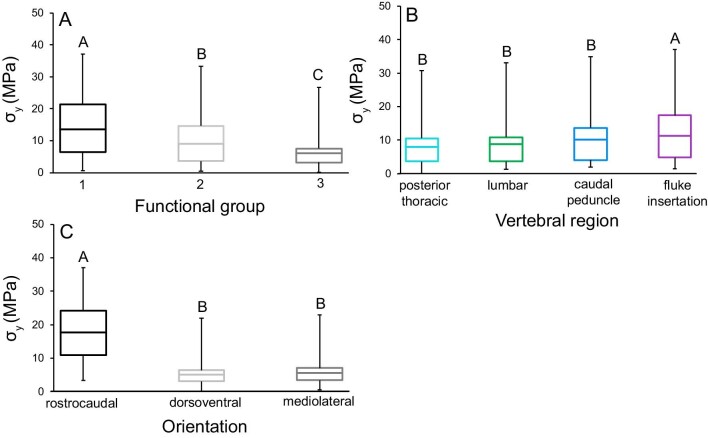
Yield strength (σ_y_; MPa) of vertebral trabecular bone varied significantly among functional groups, vertebral regions, and testing orientations. We found that bone σ_y_ was greatest **(A)** in Group 1 cetaceans (shallow diving delphinids), **(B)** at the central/posterior caudal boundary, and **(C)** in the rostrocaudal orientation. While statistical tests examined the mean σ_y_ of cubes from each vertebra from every species (binned into functional groups), these figures contain data from each mechanical test to show the true range of each property. The center bar in each box denotes the mean, boxes are the 1st and 3rd quartiles, and the whiskers are the minimum and maximum σ_y_. Tukey test results (in letters) above box and whisker plots show significant differences, with A denoting the greatest mean. Yield strengths ranged between 0.1 and 37.6 MPa. The bottom 20% of values were from dorsoventrally and mediolaterally compressed bone from all vertebral regions and species, with the exception of the Risso's, pantropical spotted, and Atlantic spotted dolphins (Group 1). The top 20% of values were from all regions of the vertebral column in delphinids (Groups 1 and 2), the overwhelming majority being from rostrocaudally tested bone.

The three-way ANOVA examining σ_y_ of cetacean vertebral trabecular bone was significant (*P* < 0.001). Functional group (*P* < 0.001), region (*P* = 0.014), and testing orientation (*P* < 0.001) were significant main effects. However, the functional group*region interaction term was not significant. Tukey *post hoc* tests of main effects revealed that the greatest trabecular bone σ_y_ was measured in Group 1, from the central/posterior caudal region and in the rostrocaudal orientation (Fig. 3; Table 2).

**Table 2:** Average (± SEM) yield strength (σ_y_), apparent stiffness (*E*_app_), and resilience (*U*_r_) of vertebrae from 10 cetacean species organized by functional group, region of the vertebral column, and testing orientation

	σ_y_ (MPa)	*E*_app_ (MPa)	*U*_r_ (J/mm^3^)
Functional group			
1	11.7 ± 0.5	204.3 ± 8.3	0.6 ± 0
2	8.3 ± 0.4	157.4 ± 8	0.4 ± 0
3	8.7 ± 0.4	160.3 ± 8.5	0.5 ± 0
Vertebral region			
Posterior thoracic	8 ± 0.4	152.9 ± 8.3	0.4 ± 0
Lumbar	8.9 ± 0.4	167.3 ± 8.8	0.5 ± 0
Caudal peduncle	10.2 ± 0.5	179.5 ± 9.1	0.6 ± 0
Fluke insertion	11.6 ± 0.6	203 ± 12.7	0.7 ± 0
Testing orientation			
Rostrocaudal	17.8 ± 0.4	327.7 ± 9	1 ± 0
Dorsoventral	5.4 ± 0.2	91.1 ± 9	0.3 ± 0
Mediolateral	5.6 ± 0.2	103.4 ± 3.5	0.3 ± 0

The output from the GLM analysis for dorsoventrally tested trabecular bone σ_y_ was not statistically significant (*R*^2^ = 0.102; *P* = 0.083). However, the output for the GLM examining bone σ_y_ tested in the mediolateral orientation was significant (*R*^2^ = 0.187; *P* = 0.007), and animal length was a significant covariate (*P =* 0.003). Vertebral process length was not significant in either GLM.

### Apparent stiffness (*E*_app_) of vertebral bone

**Fig. 4:**
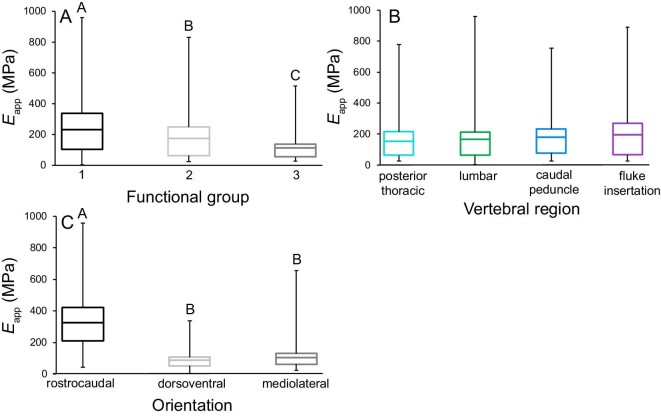
Apparent stiffness (*E*_app_; MPa) varied significantly among functional groups and testing orientations. **(A)** Bone was the stiffest (*E*_app_) in Group 1. **(B)** We found no regional variation in bone *E*_app_. **(C)** Rostrocaudally tested bone had the greatest *E*_app_. While statistical tests examined the mean *E*_app_ of cubes from each vertebra from every species (binned into functional groups), these figures contain data from each mechanical test to show the true range of each property. The center bar in each box denotes the mean, boxes are the 1st and 3rd quartiles, and the whiskers are the minimum and maximum *E*_app_ values. Tukey test results (in letters) above box and whisker plots show significant differences, with A denoting the greatest mean. Apparent stiffness ranged between 2.8 and 958.8 MPa. The bottom 20% of values were from dorsoventrally and mediolaterally compressed bone from all vertebral regions and species, with the exception of Atlantic spotted dolphins (Group 1). The top 20% of values were from all regions of the vertebral column in all species, most being from rostrocaudally tested bone.

The three-way ANOVA examining *E*_app_ was significant (*P* < 0.001), and functional group (*P* < 0.001) and testing orientation (*P* < 0.001) were significant effects (Fig. 3). Region and the functional group*region interaction term were not significant. *Post hoc* Tukey tests of significant main effects showed that the greatest bone *E*_app_ was from Group 1 animals (Fig. 4A; Table 2) and tested rostrocaudally (Fig. 4C; Table 2).

The GLM output analyzing *E*_app_ of bone tested dorsoventrally was not significant (*R*^2^ = 0.0332; *P* = 0.46). Conversely, the GLM output assessing mediolaterally tested trabecular bone demonstrated that *E*_app_ was significant (*R*^2^ = 0.134; *P* = 0.032). Here, animal length was a significant covariate (*P =* 0.013), but transverse process length was not, and no additional *post hoc* analyses were completed.

### Resilience (*U*_r_) of vertebral bone

**Fig. 5:**
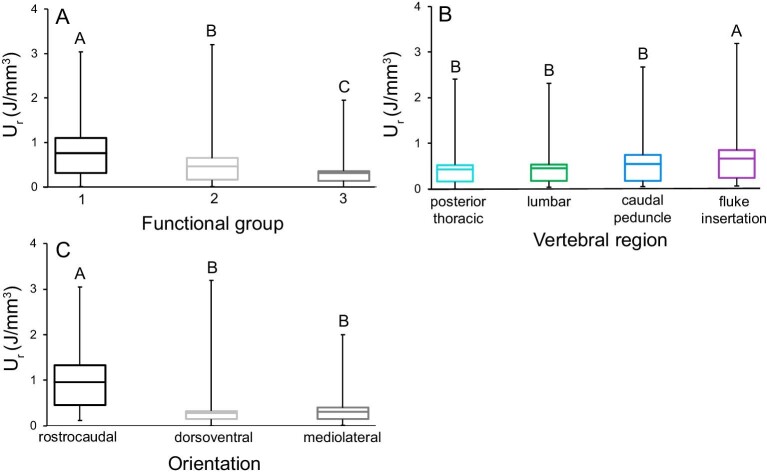
Resilience (*U*_r_; J/mm^3^) varied significantly among functional groups, vertebral regions, and testing orientations. Bone *U*_r_ was greatest **(A)** in Group 1, **(B)** at the central/posterior caudal boundary, and **(C)** in the rostrocaudal orientation. While statistical tests examined the mean *U*_r_ of cubes from each vertebra from every species (binned into functional groups), these figures contain data from each mechanical test to show the true range of each property. The center bar in each box denotes the mean, boxes are the 1st and 3rd quartiles, and the whiskers are the minimum and maximum *U*_r_ values. Tukey test results (in letters) above box and whisker plots show significant differences, with A denoting the greatest mean. Resilience ranged between 0.0004 and 5.693 J/mm^3^. The bottom 20% of values were from dorsoventrally and mediolaterally compressed bone from all vertebral regions and species. The top 20% of values were from all regions of the vertebral column in all species, with the exception of dwarf sperm whales, and the majority were from rostrocaudally tested bone.

The three-way ANOVA was significant for *U*_r_ (*P* < 0.001), and functional group (*P* < 0.001), region (*P* = 0.003), and testing orientation (*P* < 0.001) were significant main effects (Fig. 5). The functional group*region interaction was not significant. *Post hoc* Tukey tests of main effects showed that greatest bone *U*_r_ was from Group 1 animals, the central/posterior caudal region, and tested in the rostrocaudal orientation (Fig. 5; Table 2).

The GLM output examining bone *U*_r_ tested in the dorsoventral orientation was significant (*R*^2^ = 0.173; *P* = 0.013), with a significant covariate of animal length (*P* = 0.011), while vertebral spinous process length was not significant. The GLM analyzing *U*_r_ of bone tested mediolaterally did not result in a statistically significant output (*R*^2^ = 0.053; *P* = 0.27).

## Discussion

This study presents novel results on the mechanical behavior of trabecular bone from the vertebral column of cetaceans, which undergoes variable bending during caudal oscillatory swimming (Fish 1993). We used functional groups to examine the combined influences of vertebral morphology, cetacean swimming mode, and diving ecology on loading of the vertebral column. We found significant differences in vertebral trabecular bone mechanical properties among groups; where bones from Group 1 had the greatest yield strength, apparent stiffness and resilience, Group 3 had the least, and Group 2 animals had intermediate values (Figs. 3A, 4A and 5A; Table 2). Additionally, the greatest yield strength and resilience were measured from the central/posterior caudal region of all three groups, and the lowest values were from the thoracic region (Figs. 3B and 5B). Our results also demonstrated that vertebral trabecular bone was consistently the strongest, stiffest, and most resilient in the rostrocaudal orientation, which indicates that this axis is the principal direction of stress (Figs. 3C, 4C and 5C; Table 2). Finally, we compare our findings on cetacean bone mechanical properties with previous work on other mammalian species spanning terrestrial and aquatic habitats.

### Loading of the cetacean vertebral column

#### Diving ecology and swimming speed

Ecological influences such as foraging depth drive differential loading of cetacean vertebral columns. When these species descend to approximately 70 m, air is compressed within the thoracic cavity, allowing the now negatively buoyant animal to prolong sinking glides in the water column (Ridgway and Howard 1979; Skrovan et al. 1999; Williams et al. 2000). Although all diving animals must actively swim to resurface, those that habitually surpass the pivotal depth of 70 m are spending a greater proportion of time gliding than their shallow-diving counterparts and thereby potentially placing fewer demands on the vertebral column (Skrovan et al. 1999; Williams et al. 2000). We specifically investigated the influence of habitual diving ecology on vertebral trabecular bone behavior and found that shallow-diving delphinid (Group 1) bone had a greater resistance to deformation (σ_y_, yield strength), material rigidity (*E*_app_, apparent stiffness), and ability to absorb energy (*U*_r_, resilience) compared with bone from deep-diving delphinids (Group 2) and deep-diving kogiids (Group 3; Figs. 3A, 4A and 5A; Table 2).

In addition to habitual diving behavior, swimming speed may also drive loading of the vertebral column. Among cetaceans, delphinids are considered some of the most active and high-speed swimmers (Long et al. 1997; Buchholtz 2001; Marchesi et al. 2016; Marchesi et al. 2020b). Indeed, the average swimming speeds of bottlenose dolphins and pantropical spotted dolphins have been reported at about 2.8 and 2 m s^−1^, respectively, while the mean speed of a pygmy sperm whale was only 1.4 m s^−1^ (Lockyer and Morris 1987; Wood 1998; Scott and Chivers 2009). The overall greater vertebral bone apparent stiffness, resilience, and yield strength of delphinids suggest they place relatively greater mechanical demands on their vertebral columns, which may be due to their faster swimming speeds (Figs. 3A, 4A and 5A; Table 2). However, interfamilial variations in vertebral trabecular bone mechanical properties may also be influenced by phylogeny; previous work investigating trabecular bone structure has shown minor to complex differences between species, which would impact bone material behavior (Smit et al. 1997; Doube et al. 2011; Ryan and Shaw 2013; Houssaye et al. 2014). Among species examined in this study, kogiids are basal odontocetes (toothed whales), while delphinids represent the most recent radiation of this group (Chen et al. 2011; McGowen 2011). A limit to our study is that our specimens only spanned two cetacean families, and we did not conduct phylogenetic analyses. Instead, we binned delphinids and kogiids in separate functional groups (Groups 1+2 and Group 3, respectively). Future studies that incorporate a broader representation of cetacean families, and the species therein, should investigate potential phylogenetic influences on bone structure and function. However, obtaining fresh bone for mechanical testing from a phylogenetically diverse samples of these protected species may prove difficult.

#### Vertebral column morphology and differential bending

Variable bending along the axial skeleton is partially mediated by vertebral morphology; for example, disc-shaped vertebral bodies create a rigid section, while longer, spool-shaped bones denote flexibility (Pabst 1993; Long et al. 1997; Buchholtz 2001). Buchholtz (2001) found interspecific variations in the morphology of cetacean torso vertebrae and hypothesized that during swimming, dorsoventral displacement can originate anywhere between the chest (i.e., thoracic vertebrae) and caudal peduncle (i.e., anterior and central caudal vertebrae). Species in this study represent disparate ends of the flexible–rigid axial body continuum.

Although dorsoventral displacement during swimming has not been measured in kogiids, swimming style can be inferred from vertebral morphology. Similar to our vertebral measurements, Buchholtz (2001) found that a dwarf sperm whale had anterior and central torso vertebrae that were nearly equal to posterior chest vertebrae in length, width, and height, which denotes undulatory movement in this region (Table S3). This inference is supported by the dorsoventral body bending shown throughout the torso of a humpback whale, which also had nearly equal vertebral morphologies throughout the anterior and central torso. By contrast, Buchholtz (2001) showed that seven delphinids had relatively short centra throughout the anterior and central torso, and video swimming of an Atlantic white-sided dolphin (*Lagenorhynchus acutus*) in lateral view shows a rigid axial body, with movement restricted to the caudal peduncle (i.e., posterior torso).

Despite these different caudal oscillatory patterns, we surprisingly found no significant differences in regional bone mechanical properties among functional groups, although when all groups were averaged together, vertebral bone from the torso/fluke boundary was stronger and more resilient than bone from the torso (Figs. 3B and 5B; Table 2). One caveat to this conclusion: our regional sampling protocol is that lumbar vertebrae 7–9 were consistently collected from all individuals from each species. This placement is more anterior in delphinid torsos compared with kogiid torsos due to the relatively greater number of lumbar and caudal vertebral counts in delphinids (Buchholtz 2001; Gillet et al. 2019). While our protocol may have complicated our interfamilial regional comparisons of bone mechanical properties, we expect only negligible or slight biases, since centrum dimensions, and inferred regional functions, are uniform throughout the kogiid torso (Buchholtz 2001).

Bone apparent stiffness was uniform throughout the torso and at the torso/fluke boundary (Fig. 4B). These results are inconsistent with previous findings on trabecular bone mechanical properties along the vertebral column in manatees, undulatory swimmers that are generally slower than caudal oscillating cetaceans (Lockyer and Morris 1987; Wood 1998; Scott et al. 2001; Klatsky et al. 2007; Kojeszewski and Fish 2007; Ingle and Porter 2020). Adult manatee thoracic bone was stiffer than bone in lumbar and caudal regions (Ingle and Porter 2020). The stiffness of the thoracic vertebrae of manatees may be contributing to the rigidity of the anterior body, materially supporting the pachyosteosclerotic (swollen and dense) ribs, and countering air volume in the massive lungs to maintain hydrostasis (Domning and de Buffrenil 1991; de Buffrénil et al. 2010).

In the rigid-torso bottlenose dolphins and common dolphins (*Delphinus delphis*), variable bending of the axial body is mediated by stiffening of the deep tendon (Pabst 1993). Although large forces are produced by an epaxial muscle (*m. multifidus*) in the thoracic and lumbar regions, these forces are not imparted directly to the thoracic and lumbar vertebrae, and no obvious bending occurs in these regions. Instead, *m. multifidus* contractions tense the deep tendon, which has been hypothesized to act as a skeletal element for a separate epaxial muscle (*m. longissimus*) to transmit forces to the caudal peduncle, the region of highest amplitude axial displacement (Pabst 1993). This region-dependent stiffening mechanism, in concert with passive mechanisms, may alleviate the need for materially stiff vertebral bone to contribute to body rigidity in the torso (Etnier et al. 2008).

#### Anisotropic behavior of bone

The rostrocaudal orientation has been described as the principal direction of stress in both aquatic and terrestrial mammalian vertebral columns (Figs. 3C, 4C and 5C; Table 2; Mosekilde and Mosekilde 1986; Smit et al. 1997; Smit 2002; Aiyanger et al. 2014; Ingle and Porter 2020). Cetaceans load their vertebral columns in axial (rostrocaudal) compression through lift-based propulsion powered by dorsoventral oscillation of their flukes (Parry 1949; Fish 1993). We found that rostrocaudally tested bone possessed over three times greater yield strength, apparent stiffness, and resilience than dorsoventrally and mediolaterally compressed bone, confirming that the rostrocaudal orientation is the principal direction of stress on the vertebral column in these aquatic mammals (Figs. 3C, 4C and 5C; Table 2).

We hypothesized that longer spinous and transverse processes would correlate with greater vertebral trabecular bone mechanical properties in the dorsoventral and mediolateral orientations, respectively. Contrary to our prediction, we found that vertebral process lengths were consistently a nonsignificant effect in all GLM analyses (Figs. 3C, 4C and 5C; Table 2). Although the spinous processes of animals in the present study were generally longer than transverse processes, potentially providing a greater surface for muscle attachment, we found that mechanical properties of bone compressed dorsoventrally and mediolaterally were indistinguishable.

These results differed somewhat from a previous study's findings on the functional morphology of vertebrae in Florida manatees (*T. manatus latirostris*). Ingle and Porter (2020) measured no difference in *E* or *U*_r_ between dorsoventrally and mediolaterally tested bone, but they did find that mediolaterally tested bone had greater σ_y_ compared with dorsoventrally compressed samples. Manatees, which lack the complex deep tendon architecture of dolphins, have extremely long and robust transverse processes throughout their caudal vertebrae. These processes, which are the origin and insertion sites of several muscles (*m. intertransversarius coccygeus, m. sacrococcygeus ventralis medialis*, and *m. sacrococcygeus ventralis lateralis*), resist a greater amount of deformation than the much shorter spinous processes (Domning 1978; Kojeszewski and Fish 2007).

This lack of mechanical anisotropy between supporting orientations may reflect the anatomy of muscle insertions along the vertebral column. In species with relatively rigid torsos, like bottlenose and common dolphins, only a small portion of axial muscle fibers insert on spinous processes of the thoracic and anterior lumbar vertebrae, while the majority of fibers join the subdermal connective tissue sheath (SDS) and the superficial tendon for force transmission to the caudal region (Pabst 1993). We lack the same musculoskeletal descriptions for pygmy and dwarf sperm whales.

### Mechanical adaptations of fully aquatic mammalian bone

During evolutionary transitions from terrestrial habitats to a completely aquatic life, cetaceans have undergone changes in body plan, ecology, and general biology (Fish 2016). There are massive modifications in skeletal morphology that occurred from the earliest ancient whale (*Himalayacetus*) to the modern cetaceans (Bajpai and Gingerich 1998; Uhen 2010). For example, as ancient whale species began radiating into water, body shapes were increasingly streamlined, forelimbs became flippers, and hindlimbs were eventually lost. These morphological changes reduced drag, which was essential in the transition from quadrupedal locomotion to caudal oscillation (Fish 1996; Fish 2016). Evolutionary adaptations in movement and body morphology suggest differential loading of the skeleton between mammals experiencing gravitational loads on land and obligate swimmers in submerged, buoyant conditions (Currey 1984; Boszczyk et al. 2001).

**Fig. 6:**
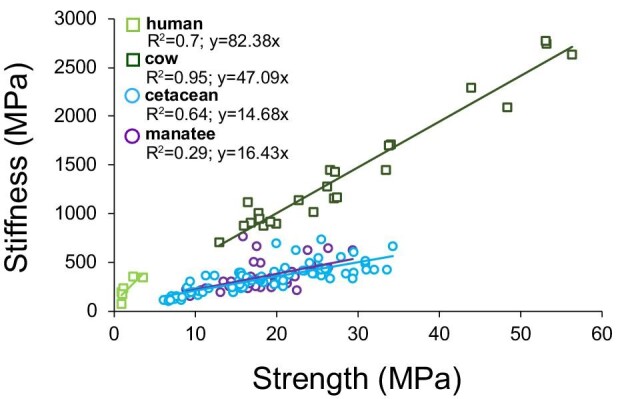
Strength–stiffness (MPa) relationships of terrestrial and aquatic mammalian vertebral trabecular bone. For any given strength, cetacean and manatee bone was less stiff compared with the bone of cows and humans. These data are from compression tests in the rostrocaudal/longitudinal orientation. Manatee (Ingle and Porter 2020) and bovine (Pilcher 2004) strength and stiffness values were each from a single dataset. The cetacean values are from this study, while human data points were averages of strength and stiffness values from multiple studies (Aiyanger et al. 2014, Banse et al. 2002, Follet et al. 2011, Matsuura et al. 2008; Slyfield et al. 2012; Yeni and Fyhrie 2001).

While limbs have been either highly modified or lost in certain mammalian groups (e.g., bat wings and aquatic mammal flippers), the vertebral column has been conserved, and the mechanical behavior and structure of its trabecular bone is an ideal indication of habitual loading throughout life (Carter and Beaupre 2001; Houssaye and Botton-Divet 2018). To understand the effects of the transition from terrestrial to aquatic locomotion on vertebral bone mechanical properties, we compared the relationship between trabecular bone strength and stiffness in cetaceans with previous findings from undulating (manatee), quadrupedal (cow), and bipedal (human) mammals (Fig. 6; Yeni and Fyhrie 2001; Pilcher 2004; Ingle and Porter 2020). Although cow and human bone strength (28.9 ± 13.1 MPa and 1.66 ± 1.08 MPa, respectively) and stiffness (1426.7 ± 631.7 MPa and 227.8 ± 106.6 MPa, respectively) varied greatly, for every given strength, land-dwelling mammals had stiffer vertebral trabecular bone than their obligate swimming counterparts (Fig. 6; Yeni and Fyhrie 2001; Banse et al. 2002; Pilcher 2004; Matsuura et al. 2008; Follet et al. 2011; Slyfield et al. 2012; Aiyanger et al. 2014; Ingle and Porter 2020). As predicted, these results suggest that relative to terrestrial mammals, fully aquatic mammalian bone has less material rigidity and bending resistance because they do not need to maintain skeletal structural integrity against gravitational loads.

## Conclusion

We investigated the vertebral bone mechanics of two cetacean families (Delphinidae and Kogiidae) with species broadly ranging in caudal oscillatory patterns and diving ecology. As the comparably more active and faster swimmers, delphinids (Groups 1 and 2) had greater vertebral trabecular bone yield strength, apparent stiffness, and resilience than kogiid species; these results suggest that delphinids habitually place greater loads on their vertebral columns compared with kogiids. Deep divers (Groups 2 and 3), who glide a greater proportion of the time than shallow-dwelling species, had relatively less strong, stiff, and resilient bone. These combined findings suggest that both phenotype (body and vertebral morphology) and behavioral ecology (habitual diving depth) drive bone mechanical behavior, although phylogeny may also play a role. Greatest yield strength and resilience at the torso/fluke boundary support previous data demonstrating that the greatest force transmission is to the posterior body during swimming. In addition, the lack of regional variation in bone apparent stiffness may reflect the predominant role of axial connective tissues in torso rigidity in cetaceans. Finally, aquatic mammals that share similar trabecular bone strength to their terrestrial mammalian counterparts may require less stiff bone due to their non-weight-bearing locomotion modes. Future research can expand upon these findings by sampling from additional cetacean families and species and investigating phylogenetic relationships among their bone mechanical properties, which could further elucidate the extent to which behavioral ecology may influence bone material behavior.

## Supplementary Material

obab036_Supplemental_Figures_and_Tables

## Data accessibility

The data underlying this article are available in Open Science Framework, and can be accessed with DOI: 10.17605/OSF.IO/YG2XS.
