# Supplementary material for: Vertebral Trabecular Bone Mechanical Properties Vary Among Functional Groups of Cetaceans
Source: Integr Org Biol. 2022 Jan 7;4(1):obab036. doi: 10.1093/iob/obab036 (PMC8832228; doi:10.1093/iob/obab036)
Supplement: obab036_Supplemental_Figures_and_Tables [file obab036_supplemental_figures_and_tables.zip › Cetacean bone mechanical properties_IOB_ESM Figures_R&R__12-03.docx]

| 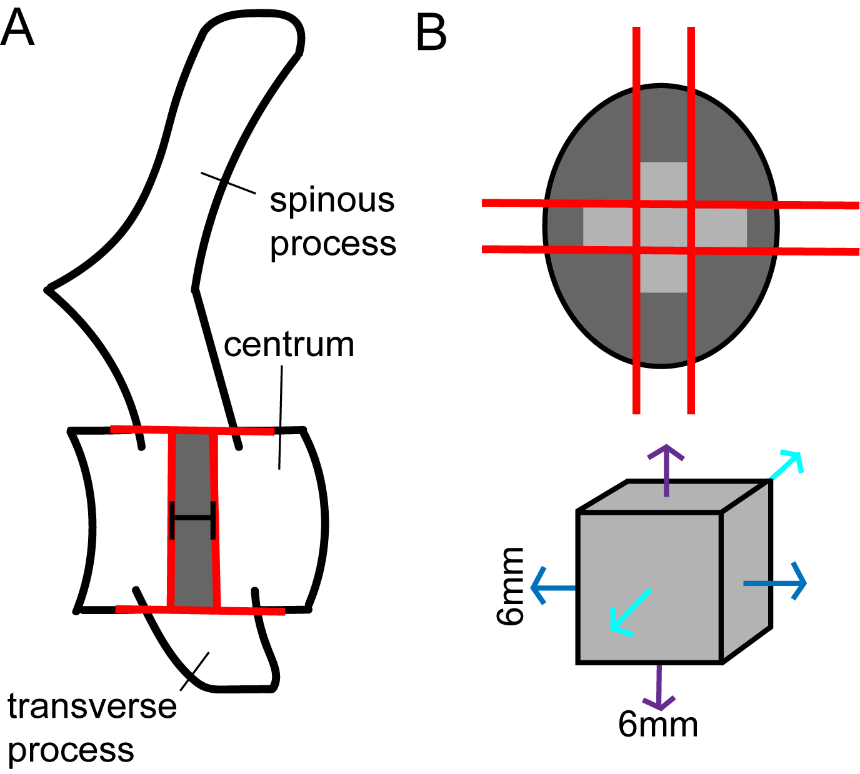 |
| --- |
| Figure S1. Bone sample preparation from vertebrae. (A) Sagittal view. Spinous and transverse processes were removed from each centrum and a 6 mm thick slice was cut from the center of it. (B) Frontal view. Five 6 mm^3^ cubes were cut from the slice of centrum. If vertebrae were too small to extract five cubes (most common in the caudal 2 region), as many cubes as possible were cut. Cubes were assigned to the rostrocaudal (RC; light blue), dorsoventral (DV; purple), and mediolateral (ML; dark blue) orientations for mechanical testing. |

| 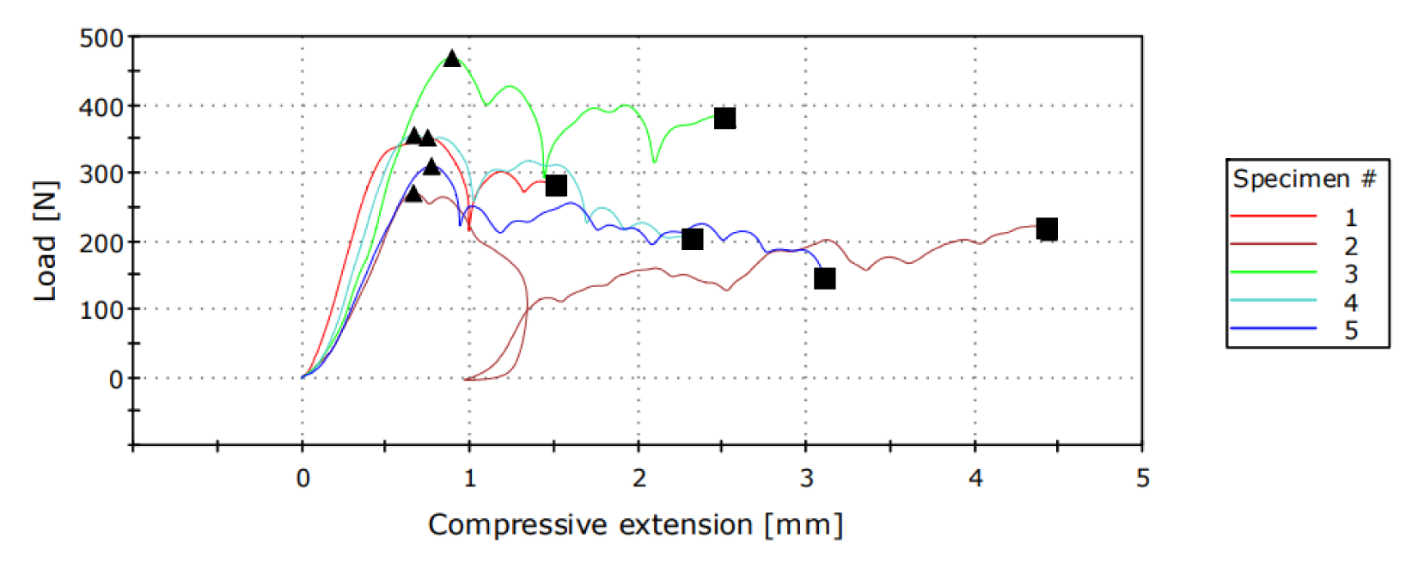 |
| --- |
| Figure S2. A load (N)-displacement (extension; mm) curve of five bone cubes (Specimen # 1-5) from a pygmy sperm whale (*Kogia breviceps*) lumbar vertebra. In this example, cubes were tested in the rostrocaudal orientation at 2 mm min^-1^. For each curve, the black triangle denotes yield (0.2% offset) and the black square denotes the termination of the test. The yield point was retrofitted from the calculation of the stress-strain curves using Bluehill Universal software. This figure was adapted from the Bluehill Universal software output. |
