# Supplementary material for: Vertebral Trabecular Bone Mechanical Properties Vary Among Functional Groups of Cetaceans
Source: Integr Org Biol. 2022 Jan 7;4(1):obab036. doi: 10.1093/iob/obab036 (PMC8832228; doi:10.1093/iob/obab036)
Supplement: obab036_Supplemental_Figures_and_Tables [file obab036_supplemental_figures_and_tables.zip › Cetacean bone mechanical properties_IOB_ESM_Tables_R&R_12-03.docx]

| Table S1. The number of bone samples extracted from each cetacean vertebra, separated by species, functional group, and vertebral column region | | | | | | | |
| --- | --- | --- | --- | --- | --- | --- | --- |
| *Species* | *Common name* | *Group* | *Vertebral column region* | | | | |
|  |  |  | *Post. thor.* | *Lumbar* | *Caud. ped.* | *Fluke ins.* |  |
| *Feresa attenuata* | pygmy killer whale | 2 | 3-4 | 3-5 | 5 | 4-5 | |
| *Grampus griseus* | Risso’s dolphin | 1 | 4-5 | 4-5 | 5 | 4-5 | |
| *Kogia breviceps* | pygmy sperm whale | 3 | 5 | 5 | 5 | 1-5 | |
| *Kogia sima* | dwarf sperm whale | 3 | 5 | 5 | 5 | 5 | |
| *Peponocephala electra* | melon-headed whale | 2 | 4-5 | 5 | 5 | 3-5 | |
| *Stenella attenuata* | Pantropical spotted dolphin | 1 | 3-5 | 3-5 | 5 | 3-5 | |
| *Stenella frontalis* | Atlantic spotted dolphin | 1 | 3-4 | 4-5 | 5 | 1-5 | |
| *Stenella longirostris* | spinner dolphin | 2 | 2-3 | 3-4 | 4-5 | 1-3 | |
| *Steno bredanensis* | rough-toothed dolphin | 1 | 3-4 | 5 | 5 | 5 | |
| *Tursiops truncatus* | bottlenose dolphin | 1 | 3-5 | 4-5 | 5 | 3-5 | |
| Post. thor. = posterior thoracic; caud. ped. = caudal peduncle; fluke ins. = fluke insertion | | | | | | | |

| Table S2. Average (± SEM) thoracic, lumbar, and caudal vertebral spinous (black text) and transverse (gray text) process lengths (mm) from 21 cetaceans placed into three functional groups | | | | | |
| --- | --- | --- | --- | --- | --- |
| *Species* | *Field ID* | *TL (cm)* | *Average vertebral process length (mm)* | | |
|  |  |  | *posterior thoracic* | *lumbar* | *caudal peduncle* |
| Functional Group 1 |  |  |  |  |  |
| *G. griseus* | 03ECWR02016 | 278 | 126.6±3.8  71.6±6.0 | 156.4±1.8  90.9±1.2 | 80.5±4.8  48.9±3.2 |
| *S. attenuata* | GW2014015A | 215 | 94.6±5.6  52.4±6.3 | 120.8±2.2  78±2.2 | 70.5±4.5  38.8±3.1 |
| *S. frontalis* | 05ECWR022014 | 213 | 100.3±6.7  60.8±6.0 | 120.6±1.4  85.9±3.0 | 90.9±4.9  54.6±5.3 |
|  | 09ECWR051013 | 211 | 102.6±5.6  65.2±6.6 | 110.4±0.5  80.7±1.7 | 80±2.0  60.2±0.4 |
| *S. bredanensis* | GW2015009D | 232 | 117.2±11.0  74.1±1.9 | 115±2.0  63.9±1.7 | 63.7±5.5  30.7±3.7 |
| *T. truncatus* | HBOI-1601-Tt | 291 | --------------  -------------- | 136.2±2.5  74.8±1.0 | 76.2±0.6  32.9±4.8 |
|  | HBOI-1703-Tt | 201 | 89.6±3.4  58.8±5.1 | 93.3±0.7  62.±0.7 | 44.3±1.6  24.7±4.8 |
|  | HBOI-1709-Tt | 281 | 146.9  107.2±8.3 | 96.1±0.9  81.2±3.4 | 88.9±3.0  40.7±4.2 |
| Functional Group 2 |  |  |  |  |  |
| *F. attenuata* | 13ECWR091960 | 198 | 84.6±1.8  58.6±5.4 | 109±0.8  73.3±6.1 | 62±0.8  40.1±3.0 |
| *P. electra* | GW2013009A | 255 | 74.9±0.9  59.2±6.5 | 103.9±0.8  65.8±2.5 | 71.6±1.5  38.8±0.3 |
|  | 22ECWR071514 | 247 | 90.8±6.2  64.7±5.9 | 108.3±4.7  68.8±2.3 | 56.5±3.0  33.1±1.8 |
|  | HBOI-1509-Pe | 223 | 89.1±3.0  69.6±2.5 | 83.7  53.4±1.4 | 53.6±0.3  29.6±0.8 |
|  | HBOI-1802-Pe | 247 | 97±4.3  68.8±13.1 | 110±1.7  80.9±1.7 | 61.7±6.1  36.5±3.1 |
| *S. longirostris* | MMRS11609 | 199 | 100±1.2  63.8±5.4 | 99.9±0.1  74.2±1.0 | 70.1±4.0  40.5±2.4 |
|  | MMRS11612 | 157 | 71.7±3.3  57.3±4.4 | 73.4±3.7  53.7±0.7 | 61.7±8.8  36.8±3.9 |
| Functional Group 3 |  |  |  |  |  |
| *K. breviceps* | GW2015003A | 205 | 115.3±6.2  50.2±5.8 | 90.6±7.5  45.2±1.4 | 34.2±2.4  31.8±7.1 |
|  | GW2013007A | 336 | ---------------  30.8±5.2 | --------------  75.4±1.9 | 111.7±12.2  62.7±4.6 |
|  | HBOI-1707-Kb | 288 | 139.1±1.8  48±3.9 | 138.6±1.8  63±2.1 | --------------  -------------- |
|  | HBOI-1801-Kb | 297 | 141.2±4.2  47.2±6.0 | 150.5±2.3  67.7±1.8 | --------------  -------------- |
|  | HBOI-1804-Kb | 303 | 150.9±2.6  54.7±7.5 | 124.7±3.4  60.2±1.0 | 45.8±4.8  23.5±2.1 |
| *K. sima* | MARS1701 | 218 | 121.3±4.3  47.7±9.2 | 91.2±7.4  62±2.3 | 64.5±4.5  49.1±4.3 |
| TL = total length. Spinous and transverse process averages (± SEM) include measurements from all vertebrae within a region (N=3 vertebrae). Note that vertebral process lengths for vertebrae in the fluke insertion region are not included because these processes were absent in most specimens. A few posterior thoracic and lumbar processes where broken and could not be measured. | | | | | |

| Table S3. Average (± SEM) centrum dimensions (mm) among regions of the vertebral column from 21 cetaceans placed into three functional groups | | | | | | | | | | |
| --- | --- | --- | --- | --- | --- | --- | --- | --- | --- | --- |
| *Species* | *Field ID* | *Region* | | *Length* | *Width* | | *Height* | | *Relative length* | |
| Functional Group 1 |  | |  |  | |  | |  | |  |
| *G. griseus* | 03ECWR02016 | | PT | 44.7±0.6 | | 50.9±0.8 | | 22.0±0.7 | | 0.9 |
|  |  | | L | 41.5±3.0 | | 54.0±0.7 | | 25.4±0.5 | | 0.8 |
|  |  | | CP | 40.3±0.3 | | 61.5±0.7 | | 31.1±2.0 | | 0.7 |
|  |  | | FI | 40.8±3.4 | | 35.9±2.4 | | 26.9±4.0 | | 1.0 |
| *S. attenuata* | GW2014015A | | PT | 33.8±0.6 | | 32.4±0.6 | | 30.7±0.6 | | 1.1 |
|  |  | | L | 29.7±0.3 | | 39.9±3.3 | | 35.2±1.2 | | 0.8 |
|  |  | | CP | 34.2±0.5 | | 43.5±1.5 | | 42.1±1.5 | | 0.8 |
|  |  | | FI | 24.3±2.3 | | 32.8±0.2 | | 32.9±2.6 | | 0.7 |
| *S. frontalis* | 05ECWR022014 | | PT | 33.6±1.4 | | 33.6±0.9 | | 32.0±0.5 | | 1.0 |
|  |  | | L | 34.9±2.5 | | 31.7±2.7 | | 36.8±0.8 | | 1.0 |
|  |  | | CP | 42.0±0.6 | | 28.9±0.1 | | 42.9±1.5 | | 1.2 |
|  |  | | FI | 23.8±4.4 | | 35.3±1.3 | | 34.0±2.6 | | 0.7 |
|  | 09ECWR051013 | | PT | 35.3±0.5 | | 34.7±1.0 | | 32.7±1.1 | | 1.1 |
|  |  | | L | 36.4±0.2 | | 28.9±0.9 | | 35.1±0.3 | | 1.1 |
|  |  | | CP | 40.8±0.3 | | 29.2±0.5 | | 40.1±0.8 | | 1.9 |
|  |  | | FI | 34.5±0.5 | | 27.3±4.3 | | 35.2±3.2 | | 1.1 |
| *S. bredanensis* | GW2015009D | | PT | 35.8±0.8 | | 33.7±0.7 | | 33.5±0.2 | | 1.1 |
|  |  | | L | 34.7±0.3 | | 36.7±1.0 | | 41.7±1.3 | | 0.9 |
|  |  | | CP | 40.1±0.8 | | 47.5±0.8 | | 49.6±1.6 | | 0.8 |
|  |  | | FI | 33.3±2.3 | | 38.1±0.5 | | 45.6±4.0 | | 0.8 |
| *T. truncatus* | HBOI-1601-Tt | | L | 44.2±0.6 | | 50.2±2 | | 46.0±0.6 | | 1.0 |
|  |  | | CP | 50.0±1.4 | | 53.7±1.3 | | 52.4±0.4 | | 0.9 |
|  | HBOI-1703-Tt | | PT | 32.9±0.6 | | 33.4±1.1 | | 30.7±0.5 | | 1.0 |
|  |  | | L | 30.8±0.6 | | 41.9±1.5 | | 38.8±0.9 | | 0.8 |
|  |  | | CP | 38.0±0.9 | | 43.6±0.3 | | 43.0±1.4 | | 0.9 |
|  |  | | FI | 31.7±2.6 | | 35.8±0.9 | | 37.4±2.7 | | 0.9 |
|  | HBOI-1709-Tt | | PT | 43.6±1.0 | | 44.1±1.2 | | 42.0±1.2 | | 1.0 |
|  |  | | L | 39.9±0.5 | | 47.0±3.3 | | 46.9±1.6 | | 0.9 |
|  |  | | CP | 45.7±1.7 | | 43.5±1.5 | | 48.4±0.3 | | 0.9 |
| Functional Group 2 |  | |  |  | |  | |  | |  |
| *F. attenuata* | 13ECWR091960 | | PT | 33.0±0.4 | | 32.1±0.8 | | 29.0±0.1 | | 1.9 |
|  |  | | L | 34.5±2.1 | | 32.3±2.1 | | 34.4±1.5 | | 1.0 |
|  |  | | CP | 43.3±0.5 | | 28.2±0.6 | | 40.9±1.4 | | 1.3 |
|  |  | | FI | 33.5±0.7 | | 33.0±0.4 | | 45.7±1.8 | | 0.9 |
| *P. electra* | GW2013009A | | PT | 41.2±0.6 | | 39.1±1.0 | | 35.5±0.6 | | 1.1 |
|  |  | | L | 34.7±1.3 | | 40.8±0.4 | | 40.5±0.5 | | 0.9 |
|  |  | | CP | 23.8±0.2 | | 45.7±0.8 | | 43.6±0.5 | | 0.5 |
|  |  | | FI | 21.8±1.9 | | 35.1±0.7 | | 34.5±1.7 | | 0.6 |
|  | 22ECWR071514 | | PT | 38.2±1.8 | | 40.5±2.2 | | 35.1±0.5 | | 1.0 |
|  |  | | L | 36.6±1.1 | | 37.1±1.0 | | 39.6±0.9 | | 1.0 |
|  |  | | CP | 44.1±0.2 | | 23.0±0.4 | | 41.0±0.7 | | 1.4 |
|  |  | | FI | 21.9±3.0 | | 31.5±1.3 | | 33.6±3.8 | | 0.7 |
|  | HBOI-1509-Pe | | PT | 33.4±1.1 | | 34.5±0.5 | | 31.5±0.4 | | 1.0 |
|  |  | | L | 35.5±3.3 | | 33.2±2.9 | | 37.8±0.4 | | 1.0 |
|  |  | | CP | 22.0±0.5 | | 39.5±1.0 | | 37.9±0.2 | | .6 |
|  |  | | FI | 23.2±2.3 | | 32.6±1.2 | | 32.4±2.9 | | .7 |
|  | HBOI-1802-Pe | | PT | 38.8±0.7 | | 41.8±0.1 | | 36.3±0.4 | | 1.0 |
|  |  | | L | 40.5±0.3 | | 35.1±0.5 | | 38.4±0.8 | | 1.1 |
|  |  | | CP | 45.1±0.2 | | 23.2±0.6 | | 42.4±0.7 | | 1.4 |
|  |  | | FI | 31.0 | | 36.3 | | 41.8 | | 0.8 |
| *S. longirostris* | MMRS11609 | | PT | 28.4±0.5 | | 29.3±0.3 | | 24.0 | | 1.1 |
|  |  | | L | 31.0±0.7 | | 23.9±0.5 | | 27.3±0.1 | | 1.2 |
|  |  | | CP | 27.0±0.7 | | 34.4±0.3 | | 32.2±0.4 | | 0.8 |
|  |  | | FI | 29.5±0.8 | | 25.3±0.7 | | 34.6±0.5 | | 1.0 |
|  | MMRS11612 | | PT | 25.2±2.0 | | 23.3±0.9 | | 22.0±0.7 | | 1.1 |
|  |  | | L | 20.7±3.7 | | 27.5±0.3 | | 25.4±0.5 | | 0.8 |
|  |  | | CP | 32.3±0.9 | | 20.8±0.6 | | 31.1±2.0 | | 1.2 |
|  |  | | FI | 23.4±3.9 | | 21.0±2.5 | | 26.9±4.1 | | 1.0 |
| Functional Group 3 |  | |  |  | |  | |  | |  |
| *K. breviceps* | GW2015003A | | PT | 45.7±1.0 | | 45.0±1.2 | | 37.5±0.7 | | 1.1 |
|  |  | | L | 53.1±0.5 | | 50.7±2.2 | | 43.0±0.5 | | 1.1 |
|  |  | | CP | 51.0±0.9 | | 49.6±0.3 | | 47.3±1.0 | | 1.1 |
|  |  | | FI | 22.9±0.5 | | 26.4±1.9 | | 24.5±1.9 | | 0.9 |
|  | GW2013007A | | PT | 64.8±2.0 | | 56.3±1.0 | | 49.0±1.3 | | 1.2 |
|  |  | | L | 82.3±1.9 | | 69.0±1.7 | | 67.6±0.8 | | 1.2 |
|  |  | | CP | 86.9±2.5 | | 73.7±0.7 | | 68.9±1.5 | | 1.2 |
|  |  | | FI | 38.0±4.0 | | 44.8±1.8 | | 43.4±3.2 | | 0.9 |
|  | HBOI-1707-Kb | | PT | 62.3±2.1 | | 55.0±2.1 | | 45.6±0.3 | | 1.2 |
|  |  | | L | 75.4±0.7 | | 63.2±1.3 | | 59.0±0.5 | | 1.2 |
|  | HBOI-1801-Kb | | PT | 62.0±1.9 | | 51.9±0.2 | | 45.1±2.0 | | 1.3 |
|  |  | | L | 70.3±1.0 | | 59.7±1.9 | | 56.3±1.2 | | 1.2 |
|  | HBOI-1804-Kb | | PT | 63.2±2.9 | | 63.6±1.1 | | 56.1±1.0 | | 1.1 |
|  |  | | L | 80.9±0.2 | | 67.0±1.7 | | 63.3±1.3 | | 1.2 |
|  |  | | CP | 68.5±2.7 | | 66.5±1.2 | | 61.3±0.3 | | 1.1 |
|  |  | | FI | 34.5±3.3 | | 45.7±2.4 | | 40.2±2.7 | | 0.8 |
| *K. sima* | MARS1701 | | PT | 46.2±0.2 | | 43.8±1.1 | | 38.0±1.2 | | 1.1 |
|  |  | | L | 50.6±0.4 | | 52.0±0.6 | | 44.8±1.0 | | 1.1 |
|  |  | | CP | 55.4±1.7 | | 51.0±0.8 | | 48.6±1.2 | | 1.1­­ |
|  |  | | FI | 33.4±3.0 | | 36.3±0.5 | | 38.0±1.2 | | 0.9 |
| PT = posterior thoracic; L = lumbar; CP = caudal peduncle; FI = fluke insertion. | | | | | | | | | | |
